# Risk factors for incisional surgical site infection after elective laparoscopic colorectal surgery

**DOI:** 10.1002/ags3.12229

**Published:** 2019-01-11

**Authors:** Keigo Chida, Jun Watanabe, Yusuke Suwa, Hirokazu Suwa, Masashi Momiyama, Atsushi Ishibe, Mitsuyoshi Ota, Chikara Kunisaki, Itaru Endo

**Affiliations:** ^1^ Department of Surgery Gastroenterological Center Yokohama City University Medical Center Yokohama Japan; ^2^ Department of Surgery Yokosuka Kyosai Hospital Yokosuka Japan; ^3^ Department of Surgery NTT East Kanto Hospital Tokyo Japan; ^4^ Department of Gastroenterological Surgery Graduate School of Medicine Yokohama City University Yokohama Japan

**Keywords:** abdominal wound closure technique, colorectal (includes small bowel/appendix), laparoscopy, postoperative complication, surgical site infection

## Abstract

**Background:**

Surgical site infection (SSI) is a common morbidity in patients undergoing colorectal surgery, and the focus of previous studies has primarily been on incisional SSI. Most reports thus far have focused on open surgery rather than on laparoscopic colorectal surgery (Lap CR). Therefore, the aim of the present study was to identify the risk factors for incisional SSI in patients undergoing elective Lap CR.

**Methods:**

This retrospective study was conducted to evaluate the occurrence and risk factors of incisional SSI for elective Lap CR. From January 2008 to June 2018, 1825 consecutive patients with a preoperative diagnosis of colorectal cancer who underwent Lap CR were analyzed at a single institution.

**Results:**

Incidence of incisional SSI was 3.3%. Postoperative hospital stay (days) was significantly longer in the incisional SSI group than in the non‐incisional SSI group (8 [6‐12] vs 10 [8‐19], *P *< 0.001). Incisional SSI were significantly associated with five operative factors: blood loss (g) (*P *< 0.014), midline wound length (mm) (*P *= 0.038), suture materials (*P *= 0.014), suture technique (interrupted vs continuous mass closure, *P *= 0.003), and organ/space SSI (*P *= 0.041). Multivariate analysis showed that continuous mass closure (odds ratio 0.290; 95% confidence interval 0.101‐0.831, *P *= 0.021) was the only factor independently associated with the incidence of incisional SSI.

**Conclusions:**

Incidence of incisional SSI was comparable to that in previous reports**.** Continuous mass closure decreased the risk of incisional SSI in elective Lap CR.

## 1 INTRODUCTION

Surgical site infection (SSI) is a common morbidity in patients undergoing colorectal surgery. The National Nosocomial Infection Surveillance (NNSI) system of the Centers for Disease Control and Prevention (CDC) introduced the concept of SSI in 1992.^1^ SSI are divided into incisional SSI and organ/space SSI; however, the focus of previous studies has primarily been on incisional SSI.^2^

Among surgical procedures, colorectal surgery is regarded as carrying a particularly high risk of SSI because of the significant bacterial load in the associated organ/space.^3^ Indeed, in open colorectal surgery, the incisional SSI rate reportedly ranges from 4.7% to 26%.^4^, ^5^, ^6^

The risk factors of incisional SSI in colorectal surgery can be classified into patient‐ and operation‐related factors. In general, the patient‐related factors are considered to play a critical role in incisional SSI, and many have been identified thus far;^5^, ^7^, ^8^, ^9^ however, most are difficult to manipulate.

Surgical techniques may be able to reduce the SSI rate, and ideal approaches have long been examined, including antimicrobial suture and methods of abdominal closure.^10^, ^11^, ^12^, ^13^, ^14^ However, these previous reports have focused on open colorectal surgery, and there are few reports of risk factors for incisional SSI with laparoscopic colorectal surgery (Lap CR). In one report that investigated such risk factors, the number of cases was as small as approximately 400.^15^, ^16^ Over the past two decades, with the widespread application of Lap CR, the incidence of incisional SSI has decreased (2.7%‐8.8%),^16^, ^17^ but incisional SSI associated with this technique remain a clinical problem to be solved.

Therefore, the aim of the present study was to retrospectively evaluate the risk factors in patients undergoing elective Lap CR.

## 2 MATERIALS AND METHODS

### 2.1 Study design

**Figure 1:**
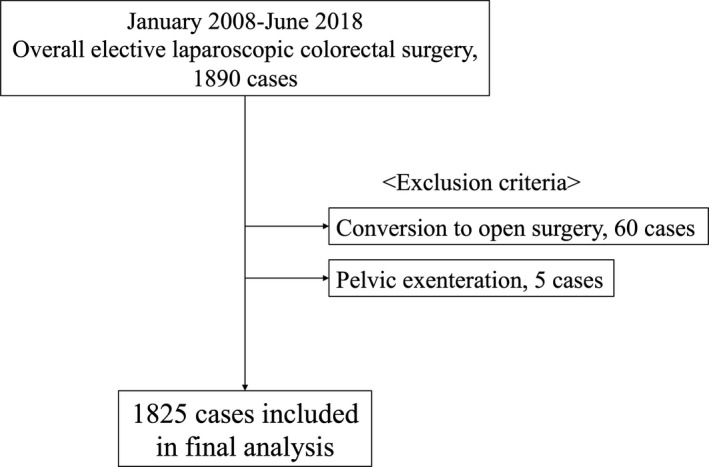
Outline of patient selection in the present study

The study protocol was approved by the Ethical Advisory Committee of Yokohama City University School of Medicine (B180400018). From January 2008 to June 2018, a total of 1890 patients who underwent elective Lap CR at Yokohama City University Medical Center were retrospectively collected. Of these, 60 cases were excluded from the analysis because of conversion to open surgery, and five were excluded because of pelvic exenteration. The remaining 1825 patients were analyzed in this retrospective study (Figure 1).

### 2.2 Variables included in the analysis

All data were retrospectively collected. Patient‐related variables analyzed were age, gender, body mass index (BMI), American Society of Anesthesiologists (ASA) class, prognostic nutritional index (PNI), comorbid disease, smoking status, preoperative chemotherapy, pathological diagnosis, maximum tumor diameter, and tumor location. Surgery‐related variables were duration of operation, amount of blood loss, surgical procedure, transfusion, use of diverting stoma, wound length, wound suture material, wound suture technique, postoperative complications, organ/space SSI, and postoperative hospital stay.

### 2.3 Antibiotic prophylaxis

During the induction of anesthesia, one dose of prophylactic i.v. antibiotics (cefmetazole 1.0 g) was given, and an additional dose was given every 3 hours during surgery and 8 hours after surgery. In cases with impaired renal function, we prolonged the dosage interval. Oral antibiotics were not used in bowel preparation.

### 2.4 Operative approach

All operations were carried out or supervised by surgeons qualified under the Endoscopic Surgical Skill Qualification System of the Japan Society for Endoscopic Surgery.^18^

Laparoscopic colorectal surgery was carried out using five ports: a 12‐mm port in the umbilical region, 5‐mm ports in the upper‐right, left, and lower‐left quadrants, and a 12‐mm port in the lower‐right quadrant. A 12‐mm umbilical trocar was used as a camera port for a rigid scope. Central vessel ligation and colon or rectum mobilization were done laparoscopically. The specimen was extracted through the umbilical port, which was extended to approximately 2‐5 cm. To avoid contamination, a wound protector was used in each case.

The skin incision was carried out with a scalpel, and the s.c. fat and linea alba were dissected by electrical cautery. Wound closure was done for the abdominal fascia using 4‐0 PDS (Ethicon, Cincinnati, OH, USA) subcuticular sutures for the skin. Prophylactic intraoperative wound irrigation with 1000 mL saline was routinely carried out before skin closure.

Suture materials used for fascia closure have changed over time as follows: April 2008‐April 2009, polyglactin 910 (Vicryl [Ethicon] JB 725, needle: CTX‐B 48 mm 1/2 circle); May 2009‐September 2012, triclosan‐coated polyglactin 910 (Vicryl plus [Ethicon] VCPB725D, needle: 48 mm 1/2 circle); October 2012‐June 2018, triclosan‐coated polydioxanone (PDS plus [Ethicon] PDP 776D, needle: 48 mm 1/2 circle).

**Figure 2:**
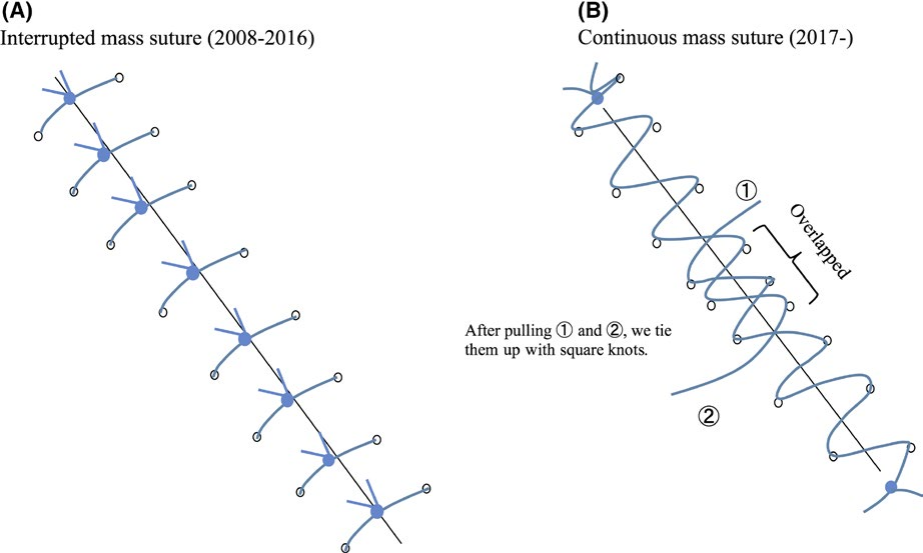
(A) illustrate interrupted suture (from 2008 to 2016) and (B) continuous closure (from 2017), respectively. Mass closure was carried out for all abdominal fascia closures

Mass closure was carried out for all abdominal fascia closures. From 2008 to 2016, interrupted sutures were used and, from 2017, continuous closure was carried out consistently (Figure 2).

### 2.5 Diagnosis of incisional SSI

All patients were monitored for postoperative incisional SSI, including superficial and deep SSI. Surgeons carried out a physical examination every day from the operating day until discharge. After hospital discharge, all patients were followed at the hospital as outpatients until day 30. Diagnosis of SSI was based on the definitions of the CDC guidelines: (i) purulent discharge with or without laboratory confirmation from the superficial incision; (ii) organisms isolated from an aseptically obtained culture of fluid or tissue from the superficial incision; (iii) at least one of the indicated signs or symptoms of infection (pain or tenderness, localized swelling, redness, or heat and superficial incision are deliberately opened by surgeon, unless the incision is culture‐negative); and (iv) a diagnosis of superficial SSI by the surgeon or attending physician.^1^ Using these definitions, incisional SSI were diagnosed in cases of such findings occurring within 30 days after surgery.

### 2.6 Statistical analyses

Primary outcome of the study was to evaluate the risk factors for incisional SSI at the midline wound. Quantitative data are expressed as median and interquartile range (IQR). We used the Mann‐Whitney *U* test to compare the median and IQR of continuous variables (such as age) and the χ^2^ test or Fisher's exact probability test to compare the proportion of categorical variables (such as gender). *P* value of 0.05 or less was considered statistically significant.

Following the univariate analysis, those variables with a *P* value less than 0.1 were selected for the multivariate analysis using the logistic regression method.

All statistical analyses were carried out with EZR (Saitama Medical Center, Jichi Medical University, Saitama, Japan), which is a graphical user interface for R (The R Foundation for Statistical Computing, Vienna, Austria). More precisely, it is a modified version of R Commander designed to add statistical functions frequently used in biostatistics.

## 3 RESULTS

A total of 1825 patients were analyzed (Figure 1). All surgical wounds were classified as clean‐contaminated (bowel was opened without spilling contents; class 2). Incisional SSI was detected in 61 out of the 1825 patients (3.34%).

### 3.1 Findings of the univariate analysis

In the univariate analysis, patients were divided into those with or without incisional SSI and compared. Table 1 shows comparisons of patient‐related characteristics. None of the variables relating to patient‐related factors was significantly associated with the development of incisional SSI. Table 2 shows comparisons of perioperative/operative‐related characteristics. Postoperative hospital stay was significantly longer in the incisional SSI group than in the non‐incisional SSI group. In addition, incisional SSI was also significantly associated with five operative factors: blood loss (g) (*P *< 0.001), midline wound length (mm) (*P *= 0.038), suture materials (*P *= 0.014), suture technique (interrupted vs continuous mass closure, *P *= 0.003), and organ/space SSI (*P *= 0.041).

**Table 1:** Patient characteristics and univariate analysis of incisional SSI

Variable	Incisional SSI (−) n = 1764		Incisional SSI (+) n = 61		*P*
	n	% or IQR	n	% or IQR	
Age, y	68	60‐75	70	64‐76	0.309
Gender, n (%)					
Male	1022	57.9	37	60.7	0.695
Female	742	42.1	24	39.3	
BMI (kg/m^2^)	22.6	20.4‐24.7	23.1	21.7‐24.8	0.087
PNI	51	47.8‐54.7	51	45.9‐55.3	0.430
ASA class					
I	314	17.8	7	11.5	0.431
II	1330	75.4	49	80.3	
III	120	6.8	5	8.2	
Diabetes mellitus, n (%)	301	17.1	10	16.4	1.000
Cardiac disease, n (%)	211	12.0	9	14.8	0.546
Hypertension, n (%)	756	42.9	29	47.5	0.512
Current or past smoker, n (%)	789	44.7	34	55.7	0.120
Neoadjuvant chemotherapy, n (%)	116	6.6	5	8.2	0.597
Histological diagnosis					
Adenocarcinoma	1706	96.7	58	95.1	0.150
Neuroendocrine tumor	39	2.2	1	1.6	
Mucinous cystadenoma	9	0.5	1	1.6	
Malignant melanoma	4	0.2	0	0.0	
Other	6	0.3	1	1.6	
Tumor site					
Colon	1232	69.8	41	67.2	0.672
Rectum	532	30.2	20	32.8	
Tumor size, mm (IQR)	32	20‐48	35	22‐51	0.430

Variables are n (%) or mean (interquartile range: IQR), unless otherwise indicated.

ASA, American Society of Anesthesiologists; BMI, body mass index; PNI, prognostic nutritional index; SSI, surgical site infection.

**Table 2:** Surgical outcomes and univariate analysis of incisional SSI

Variable	Incisional SSI (−) n = 1764		Incisional SSI (+) n = 61		*P*
	n	% or IQR	n	% or IQR	
Operative procedure					
Colectomy	1023	58.0	36	59.0	0.196
Anterior resection	607	34.4	17	27.9	
Hartmann	16	0.9	1	1.6	
ISR	59	3.3	2	3.3	
APR	59	3.3	5	8.2	
Operative time, min	181	151‐225	199	152‐240	0.163
Blood loss, g	10	5.0‐40	26	10‐68	<0.001
Transfusion					
Absent	1748	99.1	59	96.7	0.236
Present	16	0.9	2	3.3	
Stoma creation					
Absent	1362	77.2	45	73.8	0.636
Present	402	22.8	16	26.2	
Midline wound length, mm	45	40‐50	50	40‐55	0.038
Wound suture material					
Vicryl	95	5.4	3	4.9	0.014
Vicryl Plus	385	21.8	23	37.7	
PDS Plus	1284	72.8	35	57.4	
Wound suture technique					
Interrupted mass closure	1347	76.4	57	93.4	0.003
Continuous mass closure	417	23.6	4	6.6	
Postoperative complications (CD class ≥3a)					
Absent	1646	93.3	53	86.9	0.091
Present	118	6.7	8	13.1	
Organ/space SSI					
Absent	1620	91.8	51	83.6	0.041
Present	144	8.2	10	16.4	
Postoperative hospital stay, days	8	6‐12	10	8‐19	<0.001

Variables are n (%) or mean (interquartile range: IQR), unless otherwise indicated.

APR, anterior peritoneal resection; CD, Clavien‐Dindo; ISR, intersphincteric resection; SSI, surgical site infection.

### 3.2 Findings of the multivariate analysis

Table 3 shows the results of the multivariate analysis. In this analysis, only continuous mass closure was significantly associated with a decreased risk of incisional SSI (odds ratio 0.290; 95% confidence interval 0.101‐0.831, *P *= 0.021).

**Table 3:** Multivariate analysis of incisional SSI

Variable	OR^a^	95% CI	*P* a
BMI, kg/m^2^	1.080	0.997‐1.160	0.058
Blood loss, g	1.000	0.998‐1.000	0.894
Midline wound length, mm	1.010	0.986‐1.030	0.555
PDS plus	0.587	0.335‐1.030	0.063
Continuous mass closure	0.290	0.101‐0.831	0.021
Postoperative complications (CD class ≥3a)	1.250	0.449‐3.450	0.673
Organ/space SSI	1.870	0.775‐4.520	0.163

BMI, body mass index; CD, Clavien‐Dindo; CI, confidence interval; OR, odds ratio; SSI, surgical site infection.

a OR and *P* value for primary analysis (logistic regression).

## 4 DISCUSSION

The purpose of the present study was to evaluate the risk factors of incisional SSI in elective Lap CR. We showed that no patient‐related factors were associated with such SSI and that continuous mass closure significantly decreased their rate in our population.

Incidence of incisional SSI is multifactorial, and risk factors can be divided broadly into patient‐ and operation‐related factors. Patient‐related factors have been considered to play a critical role in the occurrence of incisional SSI, and various risk factors have been identified, including obesity, malnutrition, smoking, and diabetes mellitus.^5^, ^7^, ^8^, ^9^ However, in our study, no patient‐related factors were associated with incisional SSI.

Suture materials have been recognized as a potential breeding ground for infection. Sutures coated with antimicrobial compounds, such as triclosan, might reduce the rate of incisional SSI.^19^, ^20^ However, in a recent randomized control study^10^ and meta‐analysis,^21^ abdominal wall closure with triclosan‐coated sutures did not reduce the incidence of incisional SSI. Similarly, in the present study, triclosan‐coated sutures were also not associated with incisional SSI (Table 2). Although patients in the Vicryl Plus group had a slightly higher SSI rate (5.7%: 23/403), this was not higher compared with previous reports.^16^, ^17^, ^22^ Seiler et al^23^ reported that there was no difference between interrupted Vicryl and continuous PDS in terms of the incidence of SSI, but, to our knowledge, there were no reports directly comparing Vicryl Plus and PDS Plus. The concentration of triclosan in Vicryl Plus is lower than that in PDS Plus and does not provide antibacterial protection against *Escherichia coli* and *Klebsiella pneumoniae*, so these differences might have resulted in the higher SSI rate in the Vicryl Plus group compared with the PDS Plus group.

The method of closing the abdominal wall has been considered a critical aspect of incisional SSI. One such method is mass closure, which involves the closure of all layers of the abdominal wall (except for the skin) as a single structure; this technique is carried out in either a continuous or an interrupted method and significantly reduces the incidence of incisional hernia.^24^, ^25^ Therefore, in western countries, continuous mass closure has been considered safe and effective. However, in Japan, interrupted closure has been widely leveraged conventionally. In our department, based on this evidence,^24^, ^25^ continuous mass closure has been carried out consistently since 2017. Previous studies have attempted to determine the incidence of incisional hernia, and the relationship with incisional SSI has been examined as a secondary endpoint. In those studies, the incisional SSI rate was not significantly different between continuous and interrupted closure.^13^, ^26^ Nevertheless, until now, no studies have investigated the relationship between the incisional SSI rate and the closure method in cases of Lap CR. In our study, continuous mass closure significantly decreased incisional SSI, most likely for the following reasons: knots provide space in which bacteria can become enmeshed and are the most common site of sinus formation,^27^ so continuous sutures with few knots might help reduce the risk of incisional SSI in Lap CR (Figure 2).

Several limitations associated with the present study warrant mention. First, this was a retrospective analysis at a single department. Therefore, statistical analyses between the risk factors and incisional SSI were unable to determine any cause‐and‐effect relationship. Second, as SSI surveillance was judged independently by each surgeon, the standards may not have been consistent. Third, there are other known risk factors that were not evaluated but that might predispose a patient to incisional SSI, including smoking status,^28^ weight loss,^29^ intraoperative hypotension and hypoxemia,^30^ and postoperative glucose control.^31^ Moreover, the incidence of incisional hernia is an important endpoint comparing closure methods, but the aim of the present study was to evaluate risk factors of incisional SSI only and we did not examine this. In another study, we are now investigating its rate in Lap CR.

However, despite these limitations, this remains the first report of incisional SSI in a large number of Lap CR cases. Further prospective investigations will be needed to determine the risk factors of incisional SSI in elective Lap CR and the ideal closure method.

## 5 CONCLUSION

Incidence of incisional SSI was comparable to that in previous reports. Continuous mass closure of the midline fascia decreased the risk of incisional SSI in elective Lap CR.

## DISCLOSURE

Authors declare no conflicts of interest for this article.
